# Preparation of Purpurin–Fe^2+^ Complex Natural Dye and Its Printing Performance on Silk Fabrics

**DOI:** 10.3390/ma17215367

**Published:** 2024-11-02

**Authors:** Xiaojia Huang, Jie Luo, Xiangrong Wang, Xianwei Cheng, Xueni Hou

**Affiliations:** 1 College of Textile and Clothing Engineering, Soochow University, Suzhou 215006, China; 20224215013@stu.suda.edu.cn (X.H.); 17369282967@163.com (J.L.); 2 China National Textile and Apparel Council Key Laboratory of Natural Dyes, Soochow University, Suzhou 215123, China; chengxianwei@suda.edu.cn

**Keywords:** purpurin, metal ions, complex natural dyes, silk, printing

## Abstract

In order to shorten the process of textile printing with natural dyes, develop new methods, and improve the color fastness and quality of printed products, this study presents a novel approach by synthesizing a natural complex dye through the interaction between purpurin and Fe^2+^ ions, resulting in a compound named purpurin–Fe^2+^ (P-Fe). This synthesized complex dye was meticulously characterized using state-of-the-art analytical techniques, including Fourier transform infrared spectroscopy (FT-IR), ultraviolet–visible (UV–Vis) spectrophotometry, and scanning electron microscopy energy-dispersive spectroscopy (EDS). The characterization confirmed the successful complexation of purpurin with Fe^2+^ ions. The prepared complex dye P-Fe was used for the printing of silk fabric. The optimized printing process involves steaming at a temperature of 100 °C for a duration of 20 min. In comparison to fabrics printed using direct dyes, the *K*/*S* values of the fabric printed with the P-Fe complex showed a significant enhancement, with all color fastness ratings achieving grade four. Furthermore, the proportion of metal elements on the white background of the printed fabric was found to be less than 0.180%, and the level of whiteness was above 50. The application of the P-Fe dye in silk fabric printing not only streamlines the printing process but also enhances the depth and speed of the printed color, effectively addressing the issue of color transfer onto a white background, which is commonly associated with natural dyes.

## 1. Introduction

Natural dyes have historically been the cornerstone of textile dyeing [1]. Characterized by their vibrant color spectrum and superior color fastness, they have progressively replaced synthetic dyes in the domain of textile printing and dyeing [2].Contemporary society places a premium on eco-friendly practices in all facets of daily life, including food, clothing, shelter, and transportation. This shift has led to a renewed interest in natural dyes, which are celebrated for their organic origins, non-toxic properties, and environmentally benign characteristics. As a result, natural dyes are regaining prominence in textile printing and dyeing, making research and development in associated dyeing and printing technologies a focal point [3]. Silk fabrics, derived from natural fibers, can achieve authentic eco-friendly dyeing and finishing when combined with natural dyes, thereby enhancing the added value of silk products [4].

Research into dyeing silk fabrics with natural dyes primarily focuses on optimizing the dyeing process. Concurrently, investigations into silk fabric printing are primarily directed toward enhancing color fastness. This objective can be achieved through the use of mordant techniques, which involve the use of metal ions to augment the affinity between natural dyes and silk fibers, consequently enhancing color fastness [5]. For instance, Rekaby et al. [6] demonstrated that pre-mordanting silk fabrics with aluminum, ferrous ions, tin ions, and magnesium ions prior to printing with natural dyes from alkanet and rhubarb significantly improved color fastness, with ferrous ion mordanting exhibiting the most pronounced effect on fastness enhancement.

The traditional printing process involves the pre-mordanting of silk fabrics, where metal ions interact with natural dyes in the color paste with ligand groups on the fibers to form coordination structures, thereby enhancing the color yield and fastness of the printed fabric [7]. However, the conventional process is lengthy, and during washing post-printing, natural dyes that are washed off can easily form coordinate bonds with metal ions on the white background of the silk fabric, causing the color transfer to the white base and affecting the overall appearance of the printed fabric [8]. Moreover, the effluent from the mordanting treatment contains a significant amount of unbound metal ions, which has certain environmental impacts [9].

If metal ions can be preliminarily and stably complexed with natural dyes to form metal complex natural dyes [10] and then directly added to the color paste for fabric printing, it can not only shorten the process but also maximize the reaction between the dye and metal ions with silk fibers during printing, thereby reducing the impact on the white background. For instance, in Table 1, Wagner [11], it can be said that Al(III)—apigenin and Fe(II)—apigenin complexes are formed in the dyeing process, and this increases the light fastness of the dyeing. Qidi [12] prepared metal complex dyes of catechin and purpurin, which were applied to the dyeing of wool, silk, and nylon 56, showing better color fastness compared to traditional natural dyeing methods. Currently, there is limited research on the use of metal complex and natural dyes for silk fabric printing.

Addressing the issues of poor color fastness, susceptibility to color transfer, and lengthy processing times associated with traditional natural dye printing techniques, this paper explores the coordination of purpurin, derived from the madder plant, with metal ions to synthesize natural–metal complex dyes [13]: purpurin–Fe^2+^ (P-Fe). Characterization and analysis of the prepared complexes were conducted using various methods such as FT-IR, UV–Vis, and EDS [14]. Based on this, the printing performance of the complex dyes on silk fabrics was investigated, and the printing process conditions were optimized. Compared with the conventional direct printing process using natural dyes, the color depth, fastness, and white ground staining properties of the printed fabrics were evaluated. This research provides new insights into the development of more environmentally friendly printing techniques using natural dyes and enhancing the quality of natural dye-printed products.

## 2. Materials and Methods

### 2.1. Materials

Bombyx mori silk power spinning and the combination of warp and weft threads, was 3/40/44D (3 silks of 40–44 denier in diameter are intermingled as warp and weft yarns). Mulberry silk and the organization of plain weave, was the finished product of a warp density of 421 roots/10 cm and a weft density of 320 roots/10 cm, 81 g/m^2^ by Wujiang Huifang Silk Weaving Factory (Suzhou, China). Purpurin (with a purity of 97.9%) was purchased from Shanghai Yuanye Bio-Technology Co., Ltd. (Shanghai, China). Ferrous sulfate heptahydrate (99%), glacial acetic acid (99.5%), sodium carbonate (99.8%), sodium hydroxide (96%), and anhydrous ethanol (99.9%) were purchased from Chinasun Specialty Products Co., Ltd. (Suzhou, China) All the reagents used were of analytical grade. Industrial-grade guar gum and Peregal O (96%) were procured from Suzhou Youhe Textile Auxiliaries Co., Ltd. (Suzhou, China) and Jiangsu Sinofine Surfactants Technology Co., Ltd. (Suzhou, China), respectively. National standard saponin was provided by the Shanghai Textile Industry Technical Supervision Institute (Shanghai, China).

### 2.2. Preparation of Sample

In order to prepare purpurin metal complex dye, we used the following method: The molar ratio of purpurin to metal ions was set at 1:1, with the pH value adjusted to 5. The mixture was then stirred and maintained at 60 °C for 60 min. A calculated amount of purpurin was dissolved and heated to 60 °C under stirring. The pH was adjusted using acetic acid. Subsequently, a calculated quantity of iron(II) sulfate was slowly dripped into the dye solution through a peristaltic pump. The mixture was further stirred and maintained at the same temperature for the specified duration before cooling to room temperature. The resulting product was centrifuged at 6000 rpm for 15 min using a centrifuge. The precipitate was rinsed three times with a 1:1 ethanol–water solution and finally dried at 50 °C until a constant mass was achieved, yielding P-Fe.

The method for preparing P-Fe printing paste is as follows: Weigh 65 g of 10% guar gum paste into an enameled cup. Dissolve 2 g of complex natural dye, 5 g of urea, and 0.5 g of citric acid in 25 g of deionized water by heating, and successively add them to the paste in order. Stir at 1200 r/min for 60 min using a mixer until the dye is evenly dispersed in the paste. Place in a 25 °C water bath to stand and defoam for 60 min, and it is ready for use [15].

### 2.3. Printing Process

The method for preparing the pre-mordanting formula and process is as follows: the amount of mordanting agent is 5% (o.m.f), the amount of Peregal O is 0.5 g/L, and the bath ratio is 1:40. The mordanting solution was heated to 60 °C in a constant temperature water bath, and the fabric, which had been pre-wetted and squeezed dry, was immersed and mordanted for 40 min with continuous stirring. The mordanting solution was heated to 60 °C. After the mordanting was complete, the fabric was removed, rinsed, air-dried, and set aside for printing.

The printing process is given as follows: The fabric is evenly laid on a magnetic bar screen printing machine, ensuring that the fabric, screen frame, and magnetic bars are completely dry. The magnetic force of the printer is adjusted to level 20, and the machine speed is set to level 10. The prepared printing paste is poured onto the screen frame, and the magnetic bar is used to scrape the paste across the fabric twice. The fabric is then removed and placed in a drying oven at 80 °C for 5 min. The dried, printed fabric is steamed in saturated steam at 102 °C and 100% humidity for 30 min. The fabric is then washed with water at 25 °C, followed by a wash in warm water at 50 °C, with soaping (with a bath ratio of 1:40 and a standard soap flake concentration of 2 g/L at 80 °C for 20 min); a final rinse with cold water is performed at 25 °C, and the fabric is air-dried. The bath ratio for the water wash steps is 1:80.

The steaming process is as follows: The steaming process variables for the complex natural dye are temperatures ranging from 95 °C to 115 °C, with a fixed steaming time of 30 min. Additionally, the steaming time varies from 10 min to 30 min to optimize the dyeing process.

### 2.4. Testing and Characterization

#### 2.4.1. Fourier Transform Infrared (FTIR) Spectroscopy

In order to investigate the changes in functional groups used the purpurin dye subsequent to complexation with metal ions, we used infrared light spectroscopic tests, and the samples were prepared as follows: The test samples were pre-dried. Approximately 1 mg of the sample was uniformly mixed with 200 mg of potassium bromide (KBr), thoroughly ground, and then dried to remove moisture. The sample was pressed into a tablet and analyzed using a Nicolet-5700 Fourier Transform Infrared Spectrometer, Thermo Fisher Scientific, (Waltham, MA, USA). The infrared spectrum was scanned between 4000 and 400 cm^−1^ with automatic gain, and the spectral curve was plotted.

#### 2.4.2. Ultraviolet–Visible (UV–Vis) Spectroscopy Analysis

To test the UV–visible spectra of the samples, the following method was used: the solution to be tested was diluted to a specific ratio and scanned between 200 and 450 nm using a TU-1900 UV–Vis Spectrophotometer, Beijing Purkinje GENERAL Instrument Co., Ltd. (Beijing, China) to obtain the spectral curve.

#### 2.4.3. *K/S* Value and Color Characteristic Value

Four layers of printed fabric were cut and tested using an UltraScan PRO Spectrophotometer. HunterLab, (Fairfax, WA, USA). The parameters were set to a visible light range of 400–800 nm under a D65 standard light source and 10° viewing angle conditions. Different printed positions were scanned four times, and the final result was taken as the average value.

The whiteness of the white background after fabric printing was characterized by the whiteness index. The testing method was the same as above. Select the WI CIE D65/10 parameter in the machine, and the directly read value is the whiteness value [16].

*K*/*S* = (1 − R)^2^/2R − (1 − R_0_)^2^/2R_0_

$$L^{*}=116{\left(\frac{Y}{Y_{0}}\right)}^{\frac{1}{3}}-16$$

$$a^{*}=500\left[{\left(\frac{X}{X_{0}}\right)}^{\frac{1}{3}}-{\left(\frac{Y}{Y_{0}}\right)}^{\frac{1}{3}}\right]$$

$$b^{*}=200\left[{\left(\frac{Y}{Y_{0}}\right)}^{\frac{1}{3}}-{\left(\frac{Z}{Z_{0}}\right)}^{\frac{1}{3}}\right]$$

$$C^{*}=\;[{a^{*2}+b^{*2}]}^{\frac{1}{2}}$$

$$H^{*}=arctg\frac{b^{*}}{a^{*}}$$

$$W=100-\;[{{\left(100-L^{*}\right)}^{2}+a^{*2}+b^{*2}]}^{\frac{1}{2}}$$

#### 2.4.4. Color Fastness

The washing fastness of the printed silk fabrics was measured according to method A(1) of ISO 105-C10:2006 [17] on an SW-12A washing color fastness testing machine. Rubbing fastness was evaluated according to ISO105-X12:2016 [18], and light fastness was tested according to ISO105-B02:2014 [19].

#### 2.4.5. Surface Elemental Composition Analysis

A conductive adhesive can be used to stick the sample to be tested on the electron microscope table, with gold sprayed on the table containing the sample for 90 s. The TM-3030 desktop scanning electron microscope/SwiftED3000 energy spectrometer can be used to analyze the element types and contents on the sample surface.

## 3. Results

### 3.1. Characterization of P-Fe

To elucidate the structural and functional group alterations that occur upon complexation, three distinct analytical approaches were employed: Fourier transform infrared spectroscopy (FTIR), ultraviolet–visible (UV–Vis) spectroscopy, and energy dispersive spectrometry (EDS) for elemental composition analysis.

#### 3.1.1. FTIR Analysis

**Figure 1:**
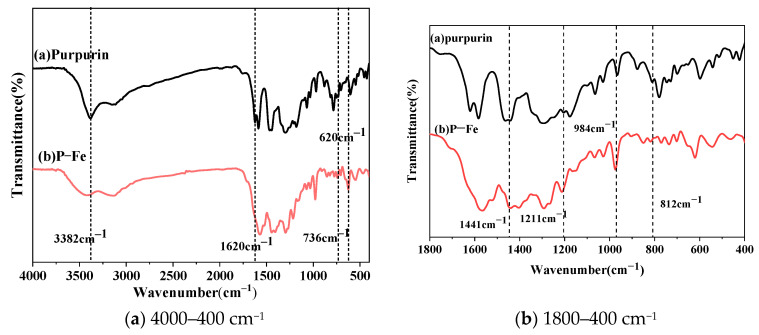
FTIR spectra of purpurin and P-Fe.

The FTIR spectra of purpurin and P-Fe are depicted in Figure 1, with the main characteristic bands detailed in Table 2.

Purpurin, an anthraquinone derivative, is primarily characterized by the presence of phenolic hydroxyl groups, carbonyl groups, and benzene rings in its molecular structure. As depicted in Figure 1, purpurin exhibits deformation and stretching vibrations associated with its phenolic hydroxyl groups, benzene rings, and carbonyl groups. Upon complexation with ferrous ions, a series of changes occur in its Fourier infrared spectrum. Notably, a new absorption peak emerges at 620 cm^−1^ in the complex dye P-Fe, which can be attributed to the stretching vibration peak formed by the coordination of ferrous ions with purpurin, resulting in an O-Fe bond. The characteristic absorption peak of the phenolic hydroxyl group of purpurin, initially at 3382 cm^−1^, and the phenolic hydroxyl group is involved in the complexation reaction between ferrous ion and purpurin, which leads to the absorption peak of the phenolic hydroxyl group being shifted to 3423 cm^−1^ [20]. The absorption peak of purpurin at 1620 cm^−1^ was the absorption peak of the carbonyl group. However, the absorption peak at 1620 cm^−1^ disappeared in P-Fe, indicating that the carbonyl group was involved in the reaction, leading to changes in its infrared properties. This suggests that the ferrous ion coordinates with purpurin to form a new complex structure.

#### 3.1.2. UV–Vis Spectra Analysis

**Figure 2:**
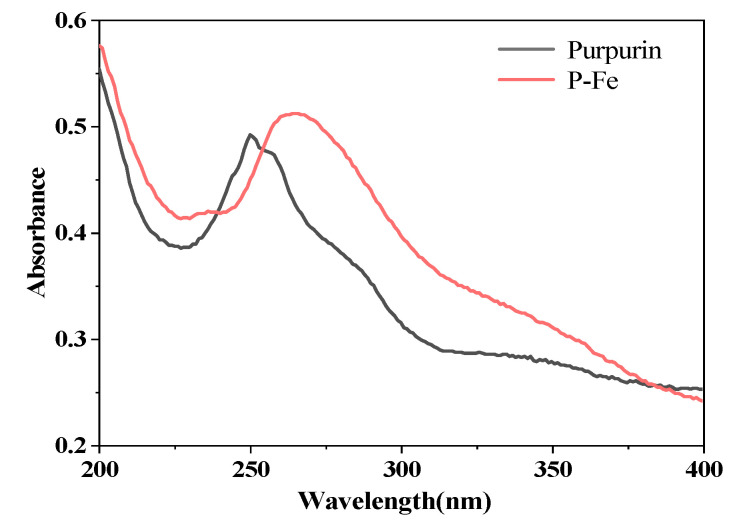
UV–Vis spectra of purpurin and P-Fe.

The purpurin solution and P-Fe solution were analyzed using a UV–visible spectrophotometer, with the resulting UV–visible spectra illustrated in Figure 2.

As can be seen from Figure 2, after the reaction of purpurin with metal ions, the maximum ultraviolet absorption wavelength of the dye solution underwent redshift. This redshift is attributed to the complexation reaction occurring at the carbonyl double bond and phenolic hydroxyl groups of purpurin, forming coordination bonds with the metal ions, which increased the planarity of the complex dye P-Fe and enhanced the conjugated system of the dye. Purpurin exhibits an absorption peak at 250 nm, but after complexation with iron ions, the original absorption peak shifted to 266 nm, which is a shift of 16 nm. Therefore, the molecular structure of purpurin changed after complexation with metal ions, indicating that a complexation reaction had occurred [21].

#### 3.1.3. Elemental Analysis

**Figure 3:**
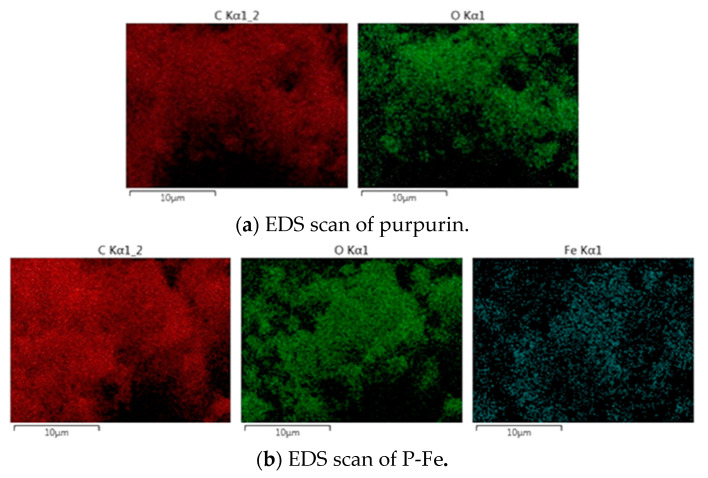
EDS scans of purpurin and P-Fe.

Elemental scans of purpurin and P-Fe were conducted, with the results depicted in Figure 3 and summarized in Table 3.

**Figure 4:**
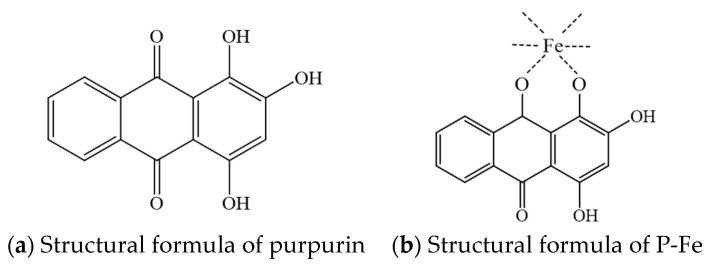
Schematic diagram of purpurin complexation with ferrous ions.

Based on the scanning results shown in Figure 3, the elemental contents of C, O, and Fe in both purpurin and P-Fe are analyzed. As can be seen from Table 3, the ratio of C atoms to O atoms in purpurin is approximately 14:5, which corresponds to the chemical structure C_14_H_8_O_5_ of purpurin. According to this structural formula, the mole ratio of C to O is 14:5. In total, 1 mol of purpurin contains 14 mol of C and 5 mol of O, so the complex dye P-Fe contains 5 mol of purpurin and 5 mol of Fe. In the P-Fe, the ratio of C atoms to O atoms and iron atoms is approximately 14:5:1, indicating that one molecule of the P-Fe contains approximately one molecule of purpurin and one Fe^2+^. From the above, it can be inferred that purpurin and ferrous ions form a 1:1 complex. Combined with the conclusions of infrared analysis, the structure of the P-Fe can be inferred, and the schematic diagram is shown in Figure 4.

### 3.2. Printing Performance of P-Fe on Silk Fabrics

The optimal printing process for P-Fe was determined by varying the steaming temperature and duration. The silk fabrics were printed under optimal printing conditions.

The printing performance of P-Fe on silk fabrics was studied by printing silk fabrics under optimal printing process conditions. This comparison facilitated an analysis of color, color fastness, and color contamination on the white background of the printed silk fabrics. This comparison helps to evaluate the performance of P-Fe in silk printing and identify any potential issues related to color quality and durability.

#### 3.2.1. Optimization of Printing Processes

Employing the single-factor experimental method, the impact of steaming temperature on the *K*/*S* value and color characteristics of P-Fe-printed silk fabrics was examined with the steaming time fixed at 15 min. Similarly, the steaming time’s effect on the *K*/*S* value and color characteristics was investigated with the steaming temperature fixed at 100 °C. The *K*/*S* value was measured at its maximum absorption wavelength was 555 nm. The results are presented in Table 4 and Table 5, respectively.

As can be seen from Table 4, the P-Fe-printed silk fabric reached a saturation value at a steaming temperature of 100 °C. Beyond this temperature, the *K*/*S* value continuously decreased with the rise in temperature. This is because the moisture content of the print paste decreased, and the diffusivity of P-Fe to the fabric was reduced. Additionally, the thermal stability of P-Fe deteriorated significantly after 100 °C, leading to the destruction of the complex structure [22]. Consequently, the coordination ability between alizarin hydroxyl and silk fabric diminished, causing a reduction in the *K*/*S* value of the printed fabric, an increase in the brightness value, and an increase in the a*, b*, and C* values. The color of the printed fabric was lighter; therefore, the optimal temperature for P-Fe printing silk fabric was chosen to be 100 °C.

As indicated in Table 5, the *K*/*S* value reached its zenith at a steaming time of 15 min. When the steaming time was more than 15 min, the *K*/*S* value no longer increased, and there were no significant changes in the brightness value, a*, b*, and C* values. This indicates that the *K*/*S* value of the P-Fe printing silk fabric reached saturation under the steaming conditions of 100 °C for 15 min.

In conclusion, the optimal steaming process for P-Fe printed silk fabric is identified as a steaming temperature of 100 °C and a steaming time of 20 min, representing a more efficient process compared to conventional steaming methods [23].

#### 3.2.2. Color Appearance of Printed Fabrics

**Figure 5:**
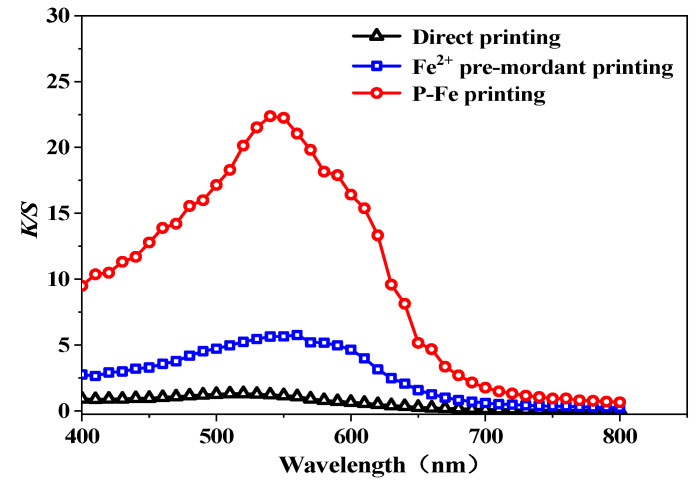
*K*/*S* value curve of purpurin direct and P-Fe printing silk fabrics.

**Figure 6:**
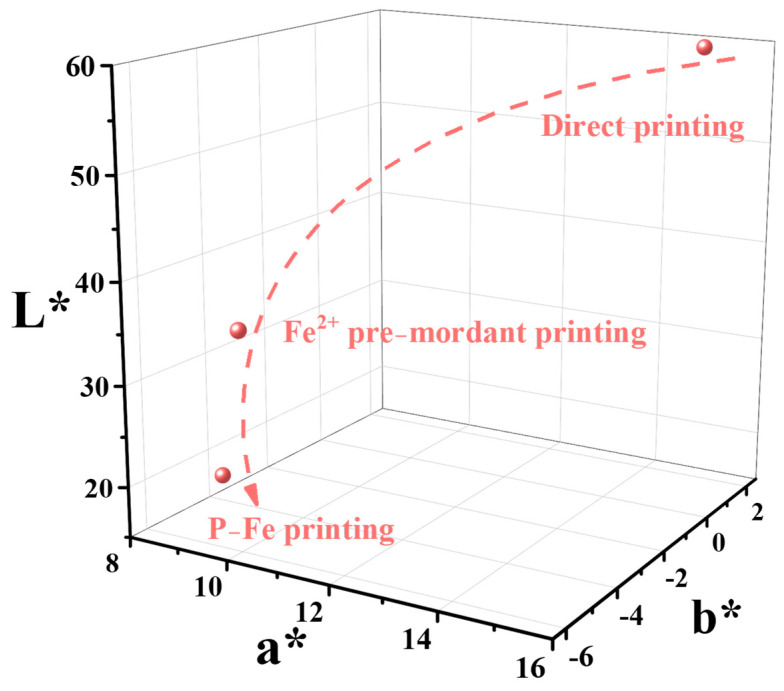
L*, a*, b* values of purpurin and P−Fe printing silk fabrics.

The color characteristics and *K*/*S* values of the directly printed, Fe^2+^ pre-mordant, and P-Fe printing silk fabrics were measured, with the results depicted in Figure 5 and Figure 6 and Table 6, respectively. The color fastness values are listed in Table 7.

From Figure 5 and Figure 6, it can be seen that, compared with the value for the directly printed silk fabric, the *K*/*S* values of the Fe^2+^ pre-mordant and P-Fe printed silk fabrics increased by 5.760 and 21.032, respectively. This indicates that both Fe^2+^ pre-mordant and P-Fe printing can enhance the color strength of the fabric after printing, with the P-Fe printing showing a greater increase. 

L* represents lightness, ranging from 100 for perfect white to 0 for absolute black [24]. The a* value represents a variation between the redness and greenness of the silk fabric (a positive value indicates red, whereas a negative value indicates green). The b* value represents a variation from the yellowness and blueness of the silk fabric (a positive value indicates yellow, whereas a negative value indicates blue) [25]. Compared with direct printing, the L*, a*, and b* values of the fabrics from Fe^2+^ pre-mordant printing and P-Fe printing decreased, indicating that the brightness and color saturation of the fabrics decreased. According to Table 6, it can be seen that the color saturation of the fabrics decreased after printing with P-Fe, and the hue of the fabrics changed considerably. The color characteristics of the fabrics from Fe^2+^ pre-mordant printing and P-Fe printing had a greater impact after printing; the effect of P-Fe printing was more pronounced, with the fabrics showing a deep purple-red color. This was due to the formation of coordination bonds between the Fe^2+^ and hydroxyl groups of purpurin, as well as hydroxyl and carboxyl groups on the silk fabric during mordant pre-mordant and complexation, which increased the dyeing rate. However, Fe^2+^ pre-mordant printing can cause staining on the white part of the printed fabric, whereas P-Fe printing has less impact on the white part of the fabric.

Washing fastness, rubbing fastness, and light fastness were measured for the directly printed, Fe^2+^ pre-mordant, and P-Fe printed silk fabrics, and the results are shown in Table 6. As shown in Table 7, compared with direct printing, the color fastness of P-Fe printing to washing and light was improved by levels of 1–2 for all fabrics.

Silk fabrics printed with P-Fe exhibited enhanced fastness due to the Fe^2+^ pre-mordant with purpurin, resulting in a greater quantity and stronger binding force of metal ions compared to silk fabrics with Fe^2+^ pre-mordant printing [26]. Consequently, the P-Fe printing silk fabrics achieved a fastness rating of grade four or more [27].

Under the optimal steaming process conditions for P-Fe, comparing the *K*/*S* value of silk fabrics after purpurin Fe^2+^ pre-mordant printing, P-Fe showed an improvement of 265.14%. The color fastness of each color reached grade four.

#### 3.2.3. Color Staining on White Background of Printed Fabrics

The whiteness of the white base portions of silk fabrics printed directly with purpurin, pre-mordanted silk fabrics printed with purpurin, and silk fabrics printed directly with the P-Fe complex dye were tested, with the results presented in Table 8. In order to further investigate the staining of the white background, surface elemental tests were conducted separately on the stained portions of the white backgrounds using three different processes. The results are presented in Table 9.

As indicated in Table 8, the original whiteness of the silk fabric was 75.025. The whiteness of the silk fabric printed with purpurin direct dye was 64.068, with minimal color contamination. This minimal contamination is attributed to residual dye from the unprinted white base during subsequent washing. When the silk fabric was pre-mordanted with Fe^2+^ prior to purpurin printing, the whiteness of the white background drastically dropped to 7.670, indicating severe staining. In contrast, the level of whiteness of the white base of the silk fabric printed directly with complex dye P-Fe was 71.130 and had the least impact on the whiteness of the fabric’s white base. 

To enhance the color depth and fastness of natural dyes during silk fabric printing, pretreatment with a mordant is commonly applied. However, this process often results in a higher residual of metal ions on the white base. During subsequent washing, the unfixed dyes rinsed from the residual color paste can combine with the residual Fe^2+^ ions, leading to a decrease in the whiteness of the unprinted white silk areas. In contrast, the use of complex dye P-Fe for printing did not cause such issues.

As shown in Table 9, when silk fabric was pretreated with Fe^2+^ and subsequently printed, the white base contained a significantly higher concentration of Fe^2+^, representing 1.562%. This increase was attributed to the formation of coordination bonds between the metal ions and the white base during the mordanting process. Conversely [28], the silk fabric printed with the complex dye P-Fe exhibited a substantially lower Fe^2+^ content, amounting to only 0.127%. This minimal residual presence was presumably due to a small fraction of the complex dye P-Fe being adsorbed onto the white base of the silk fabric during the washing phase [29].

Based on the results of color, color fastness, and white base staining, it was observed that the conventional process of mordanting before printing on fabrics improved the depth and fastness of color compared to direct printing but led to a significant decrease in the whiteness of the white base of silk fabric to 7.670, with an Fe^2+^ content of 1.562%. This represents a substantial drop from the original whiteness of 75.025, indicating severe staining. In contrast, silk fabric printed with the complex dye P-Fe showed further enhancement in both color depth and fastness, with the white base achieving a whiteness of 71.13 and an Fe^2+^ content of only 0.127%. This indicates that the use of the complex dye P-Fe for printing results in a more substantial and secure bond between the dye and the fabric, thereby improving the depth and fastness of the printed fabric’s color, with a minimal impact on the white base. Moreover, as the fabric does not require pretreatment with a mordant, the printing process was significantly streamlined [30].

## 4. Conclusions

In this study, we successfully prepared and characterized purpurin–Fe^2+^ complex dyes. Analysis using ultraviolet–visible spectroscopy and Fourier transform infrared spectroscopy identified new absorption peaks for the complex natural dye P-Fe, confirming the coordination of purpurin with ferrous ions to form a complex structure. Structural calculations and elemental content analysis revealed that purpurin predominantly formed 1:1 molar complexes with Fe^2+^, thus yielding the complex natural dye P-Fe.

The application of this complex dye to silk fabric printing was optimized. The optimal steaming parameters determined were a temperature of 100 °C and a duration of 20 min. When compared to direct printing and traditional silk fabric printing with mordant pretreatment, the fabric printed with P-Fe dye demonstrated superior color depth and fastness, with all color fastness ratings achieving grade four. The traditional silk fabric printed with mordant pretreatment had a more severe color contamination issue on the white background, with whiteness below 10 and iron ion residues exceeding 1%. In contrast, the white background of the fabric printed with P-Fe complex dye achieved whiteness above 70, and the iron ion content was less than 0.180%. This effectively addressed the problem of color contamination on the white background of natural dye printing. Furthermore, by eliminating the need for fabric mordant pretreatment, the printing process was significantly streamlined.

Purpurin–Fe^2+^ complex dyes present promising potential for the printing of silk fabrics, enhancing printing quality and color fastness while aligning with environmental and sustainability standards. Future research endeavors should focus on refining dye preparation methods, optimizing the printing process, and improving printing performance to develop more efficient and eco-friendly silk fabric printing technologies.

## Figures and Tables

**Table 1:** Dyes, metal ions, and complexes in References [11,12].

Dye	Metal Ion	Complexes	References
Apigenin	Al^3+^	Al^3+^–apigenin	[11]
Apigenin	Fe^2+^	Fe^2+^–apigenin	
Catechin	Al^3+^	Al^3+^–catechin	[12]
Catechin	Fe^2+^	Fe^2+^–catechin	
Purpurin	Al^3+^	Al^3+^–purpurin	

**Table 2:** Main characteristic bands of purpurin and P-Fe.

Characteristic Bands (cm^−1^)		Purpurin	P-Fe
3423	-OH	-	√
3382	-OH	√	-
1620	C=O	√	-
1441	C-H	√	√
1211	C-O	√	√
984	C-C	√	√
812	C-H	√	√
620	O-Fe	-	√

**Table 3:** EDS analysis results of purpurin and P-Fe.

Samples	Elemental Content (wt)/%		
	C	O	Fe
Purpurin	73.47	26.53	0.00
P-Fe	70.27	25.00	4.73

**Table 4:** Effect of steaming temperature on *K*/*S* value and color characteristic values on the P-Fe printing of silk fabrics.

Temperature/°C	*K*/*S* Value	L*	a*	b*	C*	*h*/°	Fabric Sample
95	18.01	21.99	10.10	−6.44	11.97	327.48	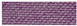
100	20.38	21.04	10.40	−6.06	12.04	329.74	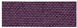
105	16.66	23.57	12.59	−7.16	14.49	330.44	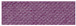
110	10.08	29.38	13.43	−8.38	15.83	328.07	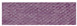
115	6.06	36.02	13.28	−8.71	15.88	326.72	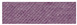

**Table 5:** Effect of steaming time on *K*/*S* value and color characteristic values on the P-Fe printing of silk fabrics.

Time/min	*K*/*S* Value	L*	a*	b*	C*	*h*/°	Fabric Sample
10	18.63	22.08	11.93	−6.96	13.82	329.74	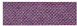
15	21.04	19.82	8.97	−5.00	10.25	330.89	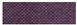
20	20.16	20.70	11.15	−4.96	12.15	336.17	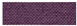
25	19.92	21.50	10.53	−6.06	12.15	330.09	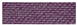
30	18.97	21.07	11.24	−5.25	12.39	335.17	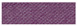

**Table 6:** Color characteristic values of printed fabrics.

Sample	C*	*h*/°
Direct printing	15.16	5.67
Fe^2+^ pre-mordant printing	11.55	329.39
P-Fe direct printing	10.26	330.88

**Table 7:** Effect of purpurin and P-Fe on the color fastness of printed silk fabrics.

Sample	Washing Fastness to Staining			Rubbing Resistance		Light Fastness
	Washing Fastness	Staining on Cotton	Staining on Silk	Dry Rubbing	Wet Rubbing	
Direct printing	3	3–4	4	4–5	4	3
Fe^2+^ pre-mordant printing	4–5	4	4	4–5	4	4
P-Fe direct printing	4–5	4	4	4–5	4	4–5

**Table 8:** Effect of P-Fe on the white background of printed silk fabrics.

Samples	White Background WI CIE [D65/10]	Fabric Sample * (White Background Section on the Right)
Silk fabrics	75.03	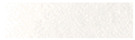
Direct printing	64.07	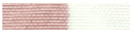
Fe^2+^ pre-mordant printing	7.67	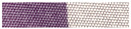
P-Fe direct printing	71.13	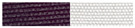

Note: * On both sides of the sample, the left side is printed fabric, and the right side is the white background staining.

**Table 9:** EDS analysis results of the white background portions of printed silk fabrics.

Samples	Elemental Content (wt)/%			
	C	N	O	Fe
Direct printing	47.01	21.32	31.67	0.000
Fe^2+^ pre-mordant printing	48.02	19.49	30.93	1.56
P-Fe direct printing	48.25	21.31	30.32	0.13

## Data Availability

The original contributions presented in the study are included in the article, further inquiries can be directed to the corresponding author.
